# Amorphous Carbon Dots and their Remarkable Ability to Detect 2,4,6-Trinitrophenol

**DOI:** 10.1038/s41598-018-28021-9

**Published:** 2018-06-27

**Authors:** Abu Bakar Siddique, Ashit Kumar Pramanick, Subrata Chatterjee, Mallar Ray

**Affiliations:** 1 Dr. M. N. Dastur School of Materials Science and Engineering, Indian Institute of Engineering Science and Technology, Shibpur, PO. Botanic Garden, Howrah, 711103 India; 2 Materials Science Division, CSIR-National Metallurgical Laboratory, Jamshedpur, 831007 India

## Abstract

Apparently mundane, amorphous nanostructures of carbon have optical properties which are as exotic as their crystalline counterparts. In this work we demonstrate a simple and inexpensive mechano-chemical method to prepare bulk quantities of self-passivated, amorphous carbon dots. Like the graphene quantum dots, the water soluble, amorphous carbon dots too, exhibit excitation-dependent photoluminescence with very high quantum yield (~40%). The origin and nature of luminescence in these high entropy nanostructures are well understood in terms of the abundant surface traps. The photoluminescence property of these carbon dots is exploited to detect trace amounts of the nitro-aromatic explosive — 2,4,6-trinitrophenol (TNP). The benign nanostructures can selectively detect TNP over a wide range of concentrations (0.5 to 200 µM) simply by visual inspection, with a detection limit of 0.2 µM, and consequently outperform nearly all reported TNP sensor materials.

## Introduction

After the serendipitous discovery of carbon dots (CDs) in 2004^1^, a substantial amount of work has been directed towards understanding these promising nanostructures and utilizing them for various applications. Among the different varieties of carbon based quasi zero-dimensional nanostructures, the amorphous variants have received much less attention compared to the more exotic crystalline structures like, graphene quantum dots, carbon quantum dots, fullerene, etc^1–9^. However, the amorphous CDs are inexpensive, easy to synthesize, water soluble and bio-compatible^2^. Studies conducted so far have revealed that the amorphous CDs have properties which are no way inferior to their crystalline counterparts^3,4^. In fact, as pointed out by Cao *et al*.^3^, the photoluminescence (PL) emissions of amorphous CDs and graphene quantum dots are similar in nearly all aspects of spectroscopic properties, including excitation dependent emission. Interestingly, the exact mechanism of PL from both the crystalline and amorphous variants of these nanostructures still remains to be completely understood^3–7^.

Amorphous CDs are synthesized by a variety of methods which usually require elevated temperatures and purification strategies^4–9^. Additionally, some passivating agents or inorganic additives are used for surface stabilization^10,11^. Approaches ranging from laser ablation to wet chemistry have been adopted for synthesis of such luminescent carbon nanostructures^2–14^. Thermal or hydrothermal treatment of carbon rich precursors like carbohydrates and polymers seem to be the most preferred routes for development of CDs^5,15,16^. Significant advancement in CD synthesis was made by Huo and co-workers^17^, when they prepared large quantities of photo-luminescent CDs through acid-induced oxidation of commercially activated carbon. Their method involved oxidation of activated carbon with nitric acid followed by surface passivation with amine terminated compounds. In this work we demonstrate that the separate step for surface protection with expensive passivating agents (like trioxa-tridecanediamine) can be avoided and strong oxidizers like HNO_3_ can be replaced by HCl to obtain amorphous and intensely fluorescent CDs. The sole reagent required is dextrose in acidic medium, which makes this method attractive for commercial applications. The water-soluble, self-passivated CDs exhibit intense room-temperature PL with a quantum yield (QY) of ~40%. Subsequently, we explore the mechanism of PL emission and demonstrate their potential in detecting trace amounts of the pollutant and explosive nitro-aromatic compound — 2,4,6-TNP.

2,4,6-TNP, also known as picric acid, is a hazardous and easily accessible nitro-aromatic compound. Though it has received much less attention than 2,4,6-trinitrotoluene (TNT), in reality, TNP is reported to possess stronger explosive ability than TNT^18,19^. Additionally, TNP is poorly biodegradable, toxic and associated with numerous human health problems^20,21^. The availability and detrimental potential of TNP, makes it an important candidate to be detected. The existing methods for detection of such compounds are spectrophotometry^22^, gaschromatography^23^, capillary electrophoresis^24^, and high performance liquid chromatography^25^. Most of these techniques are expensive and require sophisticated instruments which cannot be easily employed. Consequently, there is an ongoing effort to develop materials and techniques for detecting TNP in a simple and cost effective manner.

In 2013, Nagarkar *et al*.^26^ developed a cadmium based, 3D fluorescent metal–organic framework (MOF) for sensitive (up to 4 µM) and selective detection of TNP. In the following year Dinda *et al*.^20^ reported even better performance (selective detection of TNP up to 0.5 µM) by 2,6-diamino pyridine functionalized graphene oxide. Subsequently, Li *et al*.^27^ used blue fluorescent graphene quantum dots to develop a sensor for the analysis of TNP in water samples in the concentration range of 0.1–15 μM. Na *et al*.^28^ demonstrated that lysozyme-capped CdS quantum dots are able to detect TNP in the range of 0.5–15 μM with a detection limit of 0.1 μM. More recently, Wang *et al*.^29^ developed a Cd based MOF and utilized it as a chemosensor to detect various nitro-compounds including TNP. A larger detection range of TNP (10–600 µM, with a detection limit of 2 µM) using amine functionalized CDs has also been very recently reported by Campos *et al*.^30^. In addition to the fluorescence based methods other techniques have also been employed for sensitive detection of picric acid. Pal *et al*.^31^ used crystalline carbon dots and polypyrrole based composite films for electrical detection of TNP and achieved a detection limit of 0.14 µM.

It follows from the above discussion that some sensitive and selective techniques for detection of TNP are available. However, a major problem with some of the best techniques is the toxicity of nanomaterials. Health hazards of the Cd and other heavy metals present in MOFs are well documented^26,32^. A benign alternative based on CDs is therefore much preferred over the MOFs. In this regard, crystalline CDs and functionalized quantum dots of carbon have already shown substantial promise. Here, we demonstrate that apparently mundane, amorphous CDs having very high PL yield are excellent candidates since their synthetic strategy can result in low-cost, high output material suitable for industrial production. Additionally, we show that the amorphous CDs can detect over a much wider range (up to 200 µM) of TNP concentrations with a detection limit of 0.2 µM, which perhaps is the best performance reported so far (Supplementary Information).

## Results and Discussion

**Figure 1:**
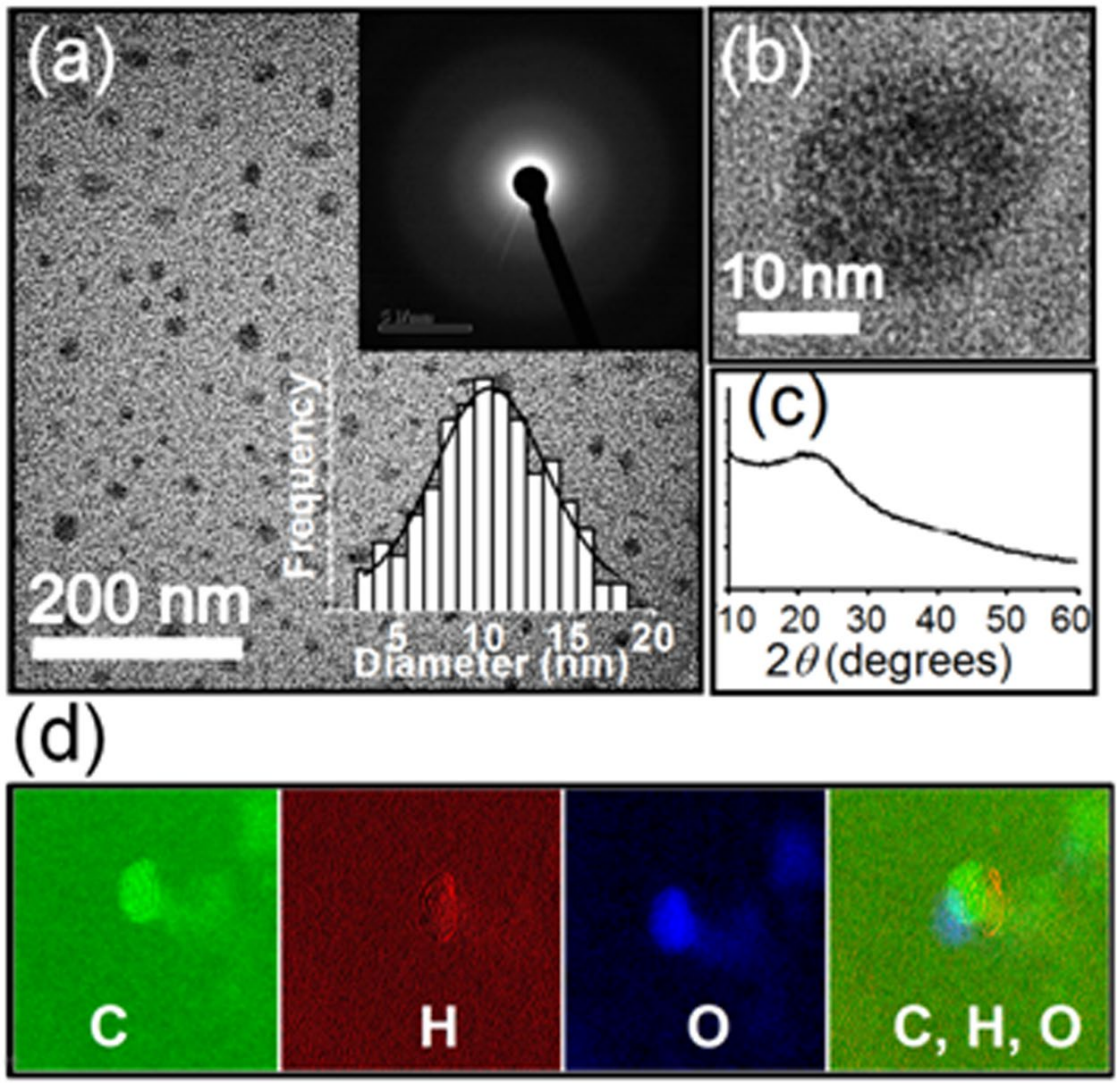
Structural features of the as-synthesized CDs. (**a**) Bright field HR-TEM image of the CDs. The top inset is the corresponding SAEDP and bottom inset shows the particle size distribution estimated from several such images; (**b**) high magnification image showing an isolated CD (no signature of any fringe); (**c**) XRD profile of the CD powder; and (**d**) left to right: elemental maps of C, H, O and their composite for a single CD obtained by EF-TEM measurements.

Ultrasonication of a mixture of dextrose and HCL followed by centrifugation and drying produces well dispersed CDs as evidenced from the high resolution transmission electron microscopy (HR-TEM) image shown in Fig. 1a. Due to the low contrast between CDs and carbon coated copper grids, bright field images of amorphous carbon based samples are seldom distinct. Nevertheless, the clear dark spots seen in the image are indicative of the formation of localized clusters of carbon atoms. The selected area electron diffraction pattern (SAEDP), shown as top inset, Fig. 1a, consists of a diffused halo devoid of any spots or rings, which is typical of an amorphous sample. These apparently amorphous clusters are nearly spherical with diameters varying from ~4–18 nm as shown in the histogram (bottom inset, Fig. 1a). No signature of fringes is present in the high magnification image shown in Fig. 1b, which captures one isolated CD. The dominant amorphous character of the CDs is also reflected in the broad hump centered around ~$2\theta ={26}^{^\circ }$ in the x-ray diffraction (XRD) profile shown in Fig. 1c. Such a hump has been widely observed in XRD patterns of amorphous carbon^35^. Elemental maps of C, H and O obtained by energy filtered TEM (EF-TEM) measurements are shown in Fig. 1d. The EF-TEM images clearly reveal the clustering of carbon atoms which is manifested by a brighter contrast in the elemental C-map. The maps of the other two elements – H and O, show their distribution, which appears to be concentrated on the surface surrounding the carbon cluster. A cluster of carbon atoms surrounded by H and O is clear from the composite mapping of all three elements, shown in the extreme right panel of Fig. 1d. Therefore, the micro-structural features of the synthesized CDs suggest that the one-step method leads to the formation of amorphous clusters of well-dispersed carbon nanostructures surrounded by O and H.

**Figure 2:**
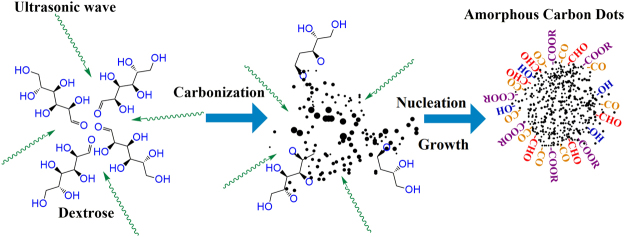
Schematic illustration of the mechanism of formation of self-passivated CDs from dextrose.

In the last few years a significant amount of work has been dedicated to understand the mechanism of formation of CDs^16,36,37^. Nearly all the proposed mechanisms of bottom-up synthesis hinge of the following steps: (i) pyrolysis of carbon rich precursors at elevated temperatures; which leads to (ii) carbonization and nucleation; followed or accompanied by (iii) surface passivation with stabilizing agents. Tang *et al*.^36^ suggested that any carbohydrate containing C, H, and O in the ratio of 1:2:1, where H and O exist in a form that allows dehydration under hydrothermal conditions, can be used for preparation of CDs. We show that carbonization of carbohydrates can be achieved through simple ultrasonic agitation at room temperature. As shown in the Fig. 2, simple mechanical agitation leads to carbonization followed by nucleation of carbon. The growth of amorphous clusters of carbon is assisted by diffusion of other molecules towards the particle surface resulting in the formation of CDs which are self-passivated with H and O related functional groups.

Surface passivation and functionalization of CDs are of paramount importance since fluorescence from these nanostructures is believed to be largely linked to the surface character^38^. Effective surface passivation is considered to be an essential step in order to produce CDs with high fluorescence yields^39^. We have nonetheless obtained highly luminescent CDs without any additional step involving external surface passivation.

**Figure 3:**
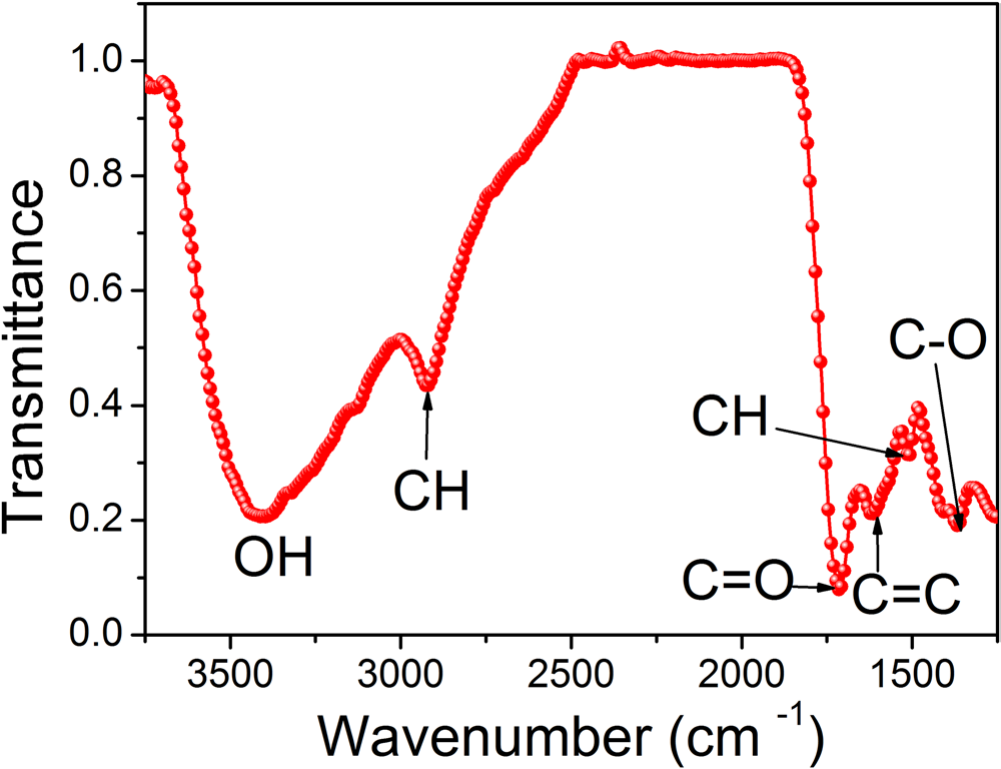
FTIR spectrum showing carbonisation of dextrose into self-passivated CDs.

In an attempt to form an idea of the bonds present in the synthesized CDs, Fourier transform Infra-red (FTIR) spectroscopy was performed and is shown in Fig. 3. The FTIR spectrum displays several peaks related to the surface bonds of CDs. The absorption band spanning from 3640 to 3160 cm^−1^ correspond to stretching vibrations of the OH bond^40^. The presence of such bonds is possibly responsible for imparting hydrophilicity and consequent water dispersibility of the CDs. The bands peaking at 1760 cm^−1^, 1680 cm^−1^ and 1150 cm^−1^ may be assigned to the stretching vibrations of C=O, C=C and C-O, respectively^41^, suggesting once again that the surface of carbon clusters are passivated by surface groups that are spontaneously derived out of the carbonization process of dextrose.

**Figure 4:**
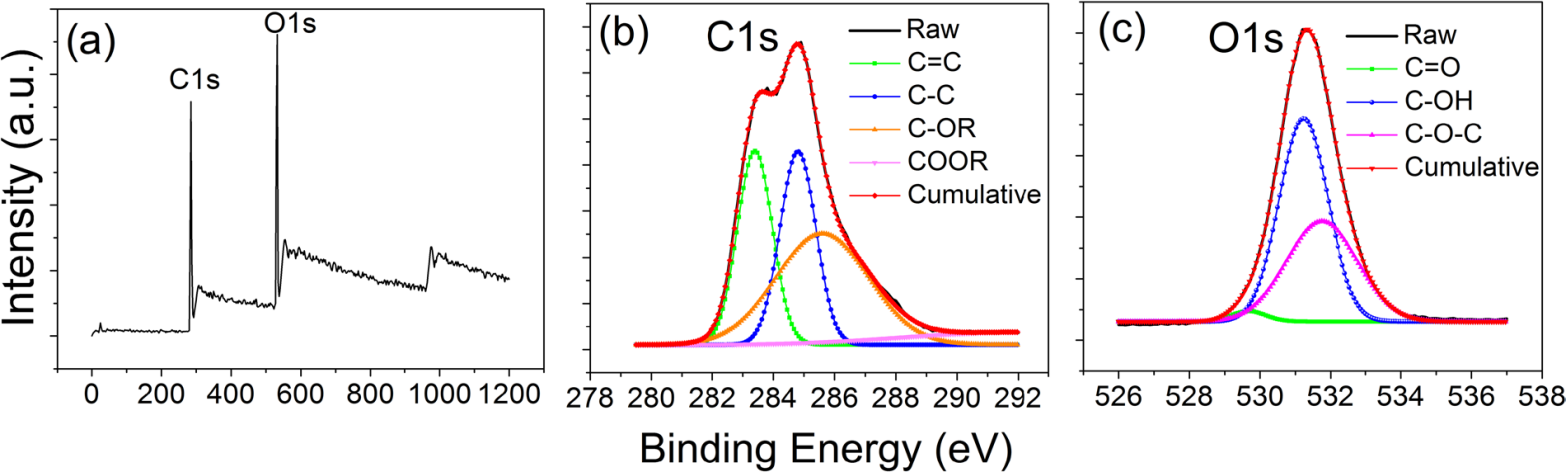
(**a**) XPS wide scan spectrum of the synthesized CDs; (**b**) C1s; and (**c**) O1s spectra of the as-prepared CDs.

X-ray photoelectron spectroscopy (XPS) measurements were carried out to further investigate the composition and surface groups of the CDs. Two strong peaks at 285.5 eV and 532.5 eV are detected in the wide-scan XPS spectrum as shown in Fig. 4a, which are attributed to oxygen and carbon, respectively^42^. The de-convoluted C1s spectrum (Fig. 4b) has four components, corresponding to C=C (*sp*^2^ carbon) at 283.2 eV, C-C (*sp*^3^ carbon) at 284.7 eV, C-OR and COOR at 285.4 eV and 289.5 eV, respectively^42–44^. The measured O1s spectrum (Fig. 4c) can be de-convoluted to three components peaking at 529.6, 531.1 and 532 eV, which are due to the C=O, C-OH and C-O-C groups, respectively. Hence, the XPS data allows us to find out the surface composition, although the relative composition of the core of the CDs, which are evidently made of C=C, cannot be obtained. Importantly, the results of XPS and FTIR reinforce each other and affirm the proposition of spontaneous self-passivation of the CDs.

**Figure 5:**
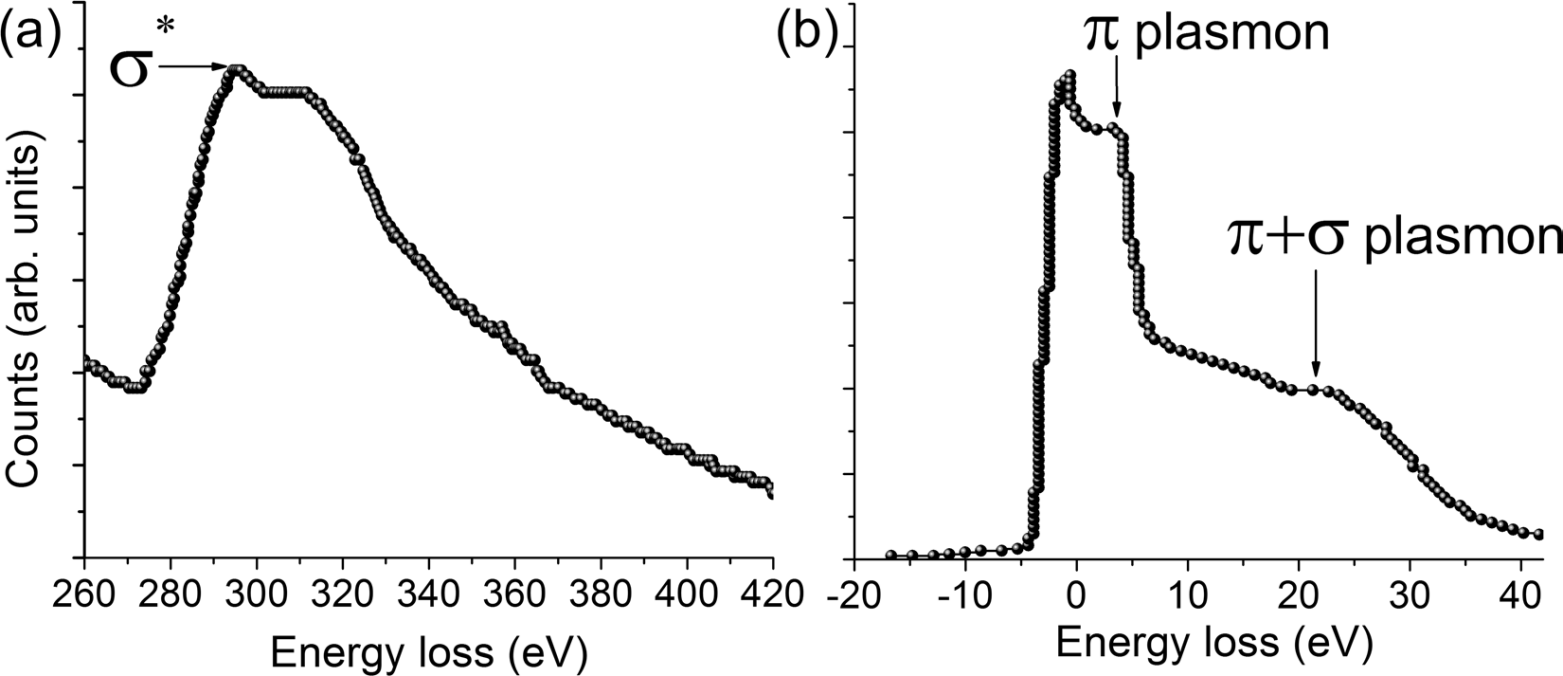
EELS spectra of the self-passivated amorphous CDs: (**a**) carbon K-edge ELNES spectrum and (**b**) low-energy-loss spectrum.

In an attempt to strengthen our understanding of the structure of the CDs, electron-energy loss spectroscopy (EELS) was performed. Figure 5a,b, show the carbon K-edge and the low-energy-loss EELS spectra of the CDs, respectively. Sufficient care was taken and the samples were tested before and after the EELS measurements to ensure protection from electron-beam induced damage and possible contamination. The carbon-K energy-loss near edge structure (ELNES) spectrum in Fig. 5a, exhibits a peak at 292 eV along with a higher energy shoulder like feature appearing at ~311 eV. The band peaking at 292 eV corresponds to 1 *s* → σ^*^ transition and is consistent with ELNES spectrum of amorphous carbon^45^. A typical and distinctive feature of graphitic carbon is a sharp peak at the onset (~285 eV) of the σ^*^ band^36,46,47^. Muller *et al*.^48^ have shown that the absence of this pre-peak (at ~285 eV) is a feature of fully amorphous carbon or graphitic structures with sizes less than 1.5 nm. Furthermore, the shoulder appearing after σ^*^ band is reported to be sensitive to the long range order^49^. A broad, plateau-like feature observed in the profile of our sample is due to the occurrence of damaged ring structures and absence of any long-range order^50^.

Figure 5b is the low-loss EELS spectrum of the CDs. The peak at 0 eV is due to the elastically scattered (or un-scattered) electrons. The peak appearing at ~5 eV is the π plasmon peak, while the π + σ bulk plasmon peak manifests as a broad hump peaking at 21.6 eV. These results are in excellent agreement with both theoretical and experimental findings reported for amorphous carbon^51–53^. The approximate density of the CDs estimated from the bulk plasmon energy peak position (21.6 eV) is found to be 1.5 g/cc, which is consistent with the reported density of graphite-like amorphous carbon (Supplementary Information)^52,54^.

Nearly all reports published so far suggest that PL from unpassivated or self-passivated CDs is usually low with a QY less than 10%^55,56^. Perhaps, the only exception is a work reported by Shen *et al*.^57^ where they showed that hydrothermally prepared crystalline CDs may have QYs as high as 40.5%, if they are treated with NaBH_4_, so as to dramatically increase surface defects. However, such studies on correlation of surface treatments and luminescence are primarily based on crystalline CDs^58,59^. In amorphous nanostructures empty or densely packed local atomic defects are abundant. Additionally, the high-entropy disordered nature of the entire structure produces multiple luminescence centers which contribute to intense PL. Despite enormous efforts in understanding the mechanism of luminescence from carbon nanostructures, a consensus about the exact mechanism is still awaited. Earlier reports in this regard focused primarily on quantum size effects and provided evidences in support of quantum confinement^60,61^. Later studies have revealed that emissive traps and surface groups are primarily responsible for bright PL from carbon nanosystems. The fact that both crystalline and amorphous CDs exhibit very similar PL characteristics indicates that defects/traps related emissive centers and surface states play dominant roles in light emission from CDs.

**Figure 6:**
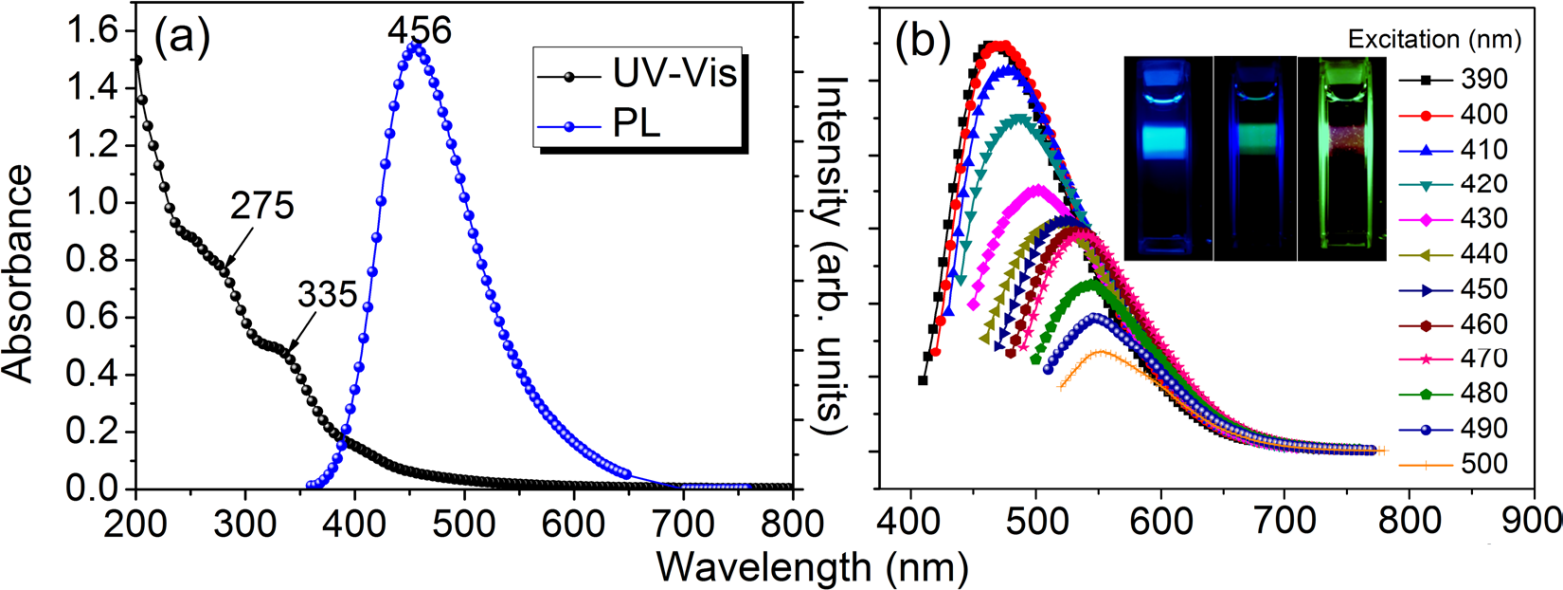
(**a**) UV-Vis and PL spectra of the CDs; and (**b**) excitation dependent PL of CDs with the inset photographs showing intense blue-green-red emissions from colloidal CDs under different excitation energies.

The PL and the UV-visible absorption profiles of the synthesized CDs are shown in Fig. 6a. The shoulders appearing at 275 nm and 335 nm in the absorption spectrum are typical of colloidal CDs absorption characteristics^39,62^. The origin of these features are related to *π* electron transitions in C=C and oxygen-containing functional groups, respectively. The shoulder at ~275 nm is probably due to π-π* transition of C=C and the absorption hump at ~335 nm possibly corresponds to n-π* transition of the C=O bond. The PL spectra on the other hand show that the dominant emissions from the CDs are in the blue-green region, which can be tuned by changing the excitation energy as shown in Fig. 6b. The photographs of the luminescent colloids exhibiting blue-green-red emissions under different excitation energies are shown as inset, Fig. 6b. It is evident that the yield is maximum in the blue-green region corresponding to UV-blue excitation and decreases with decreasing energies. The QYs for PL emission corresponding to 330 nm excitation was calculated by method proposed by Pålsson and Monkman^33^, and de Mello *et al*.^34^ and was found to be 39.5% (Supporting Information). The intense blue-green emission is also evident from the photograph of the glowing colloids. To the best of our knowledge this is the first report of such high yield self-passivated, amorphous CDs prepared at room temperature.

Nearly all variants of carbon nanostructures exhibit excitation dependent multicolour luminescence – a feature that has been widely discussed in literature for crystalline as well as amorphous samples^12,63^. Size-controlled synthesis of CDs has also been reported in literature, but, unlike semiconductor dots, tuning of PL colour by controlling the size of CDs has been challenging^3,4^. As observed by us, the as-synthesized CDs have strong PL from blue to green. Optimal emission in longer wavelength regions, though observed, is very rare for colloidal CDs^60,64,65^. Additionally, the PL bands of CDs, as seen from Fig. 6b and as reported by several other groups^66–68^, is usually pretty broad, indicating inhomogeneous chemical structure and diverse PL centers. Based on the above observations we are strongly inclined to believe that PL from CDs are essentially for emissive traps and surface functional groups. The excitation dependence of PL emission arises from the fact that at a given wavelength only certain emissive sites can be excited. Increase in excitation energy correspondingly increases the number of sites since higher energy traps can now be excited. This affects a consistent blue shift of the PL maxima accompanied by an increase in intensity with excitation energy. Thus, PL spectra of amorphous CDs also reflect the distribution of emissive centers present in the sample. In addition, we believe, discretization of phonon density of states in nanostructures also contributes towards the observed excitation dependence of emission energy. It is well known that in colloidal nanostructures the solid-liquid boundaries act as potential barriers for phonons, resulting in phonon confinement within the nanostructures^69^. The photo-excited carriers in such systems relax by emitting phonons corresponding to some discrete states. Consequently, the emission energy becomes dependent on the excitation energy. However, a detailed investigation on this particular aspect is a separate study and is being presently pursued. At this stage we think distribution of emissive trap states and discretization of phonon energies play a combined role for excitation dependence of emission from CDs.

**Figure 7:**
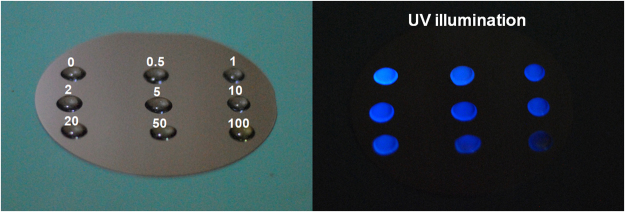
PL quenching of luminescent CDs by TNP addition. Left panel is the ambient light photograph of different concentrations of TNP added CD colloid droplets taken on a Si wafer. The numbers indicate the micro-molar concentrations of TNP in the respective drops. The right panel is the photograph of the same under UV illumination.

As a potential application of these CDs, we investigated the efficacy of these nanostructures in sensing the explosive nitro-compound — 2,4,6-TNP. A remarkable quenching of luminescence detectable with the unaided eye, as seen in the photographs (Fig. 7), was observed when trace amounts of TNP was added to the CD colloids. The visible bright blue luminescence vanishes immediately upon addition of TNP solution. Detection of the presence of TNP in solution, simply by visual inspection, was possible up to a concentration of 0.5 µM.

**Figure 8:**
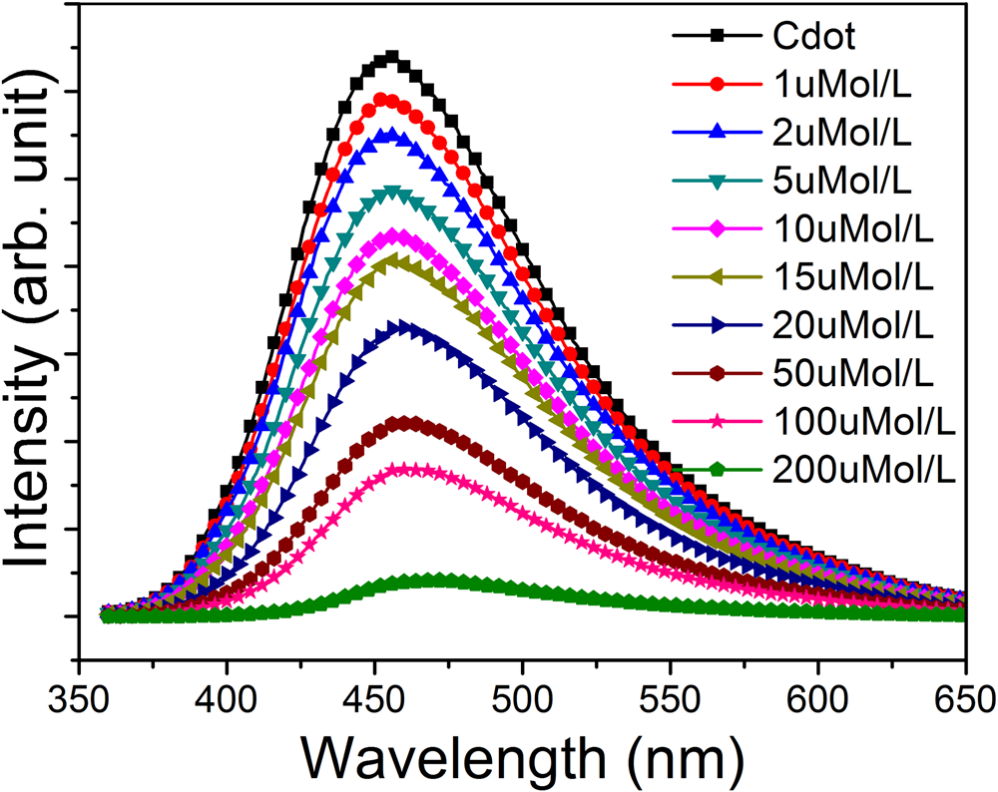
Variation of PL intensity profiles at various concentrations of TNP additions to the colloidal CDs (all concentrations are not shown to maintain clarity).

To explore the quantitative sensitivity of CDs in detecting TNP, fluorescence-quenching titrations were performed with incremental addition of TNP over a wide range of concentrations between 0.5 to 200 µM in 15 mL aqueous solution of CDs. A plot of the PL intensity profiles are shown in Fig. 8 and variation of PL maxima with different concentrations of TNP additions are shown in Figure S2 in the supplementary material. These plots, demonstrate that the variation is linear over a wide range (0.5 to 50 µM), while at higher concentrations ( > 50 µM) the intensity decreases exponentially with TNP addition (Supplementary Information).

**Figure 9:**
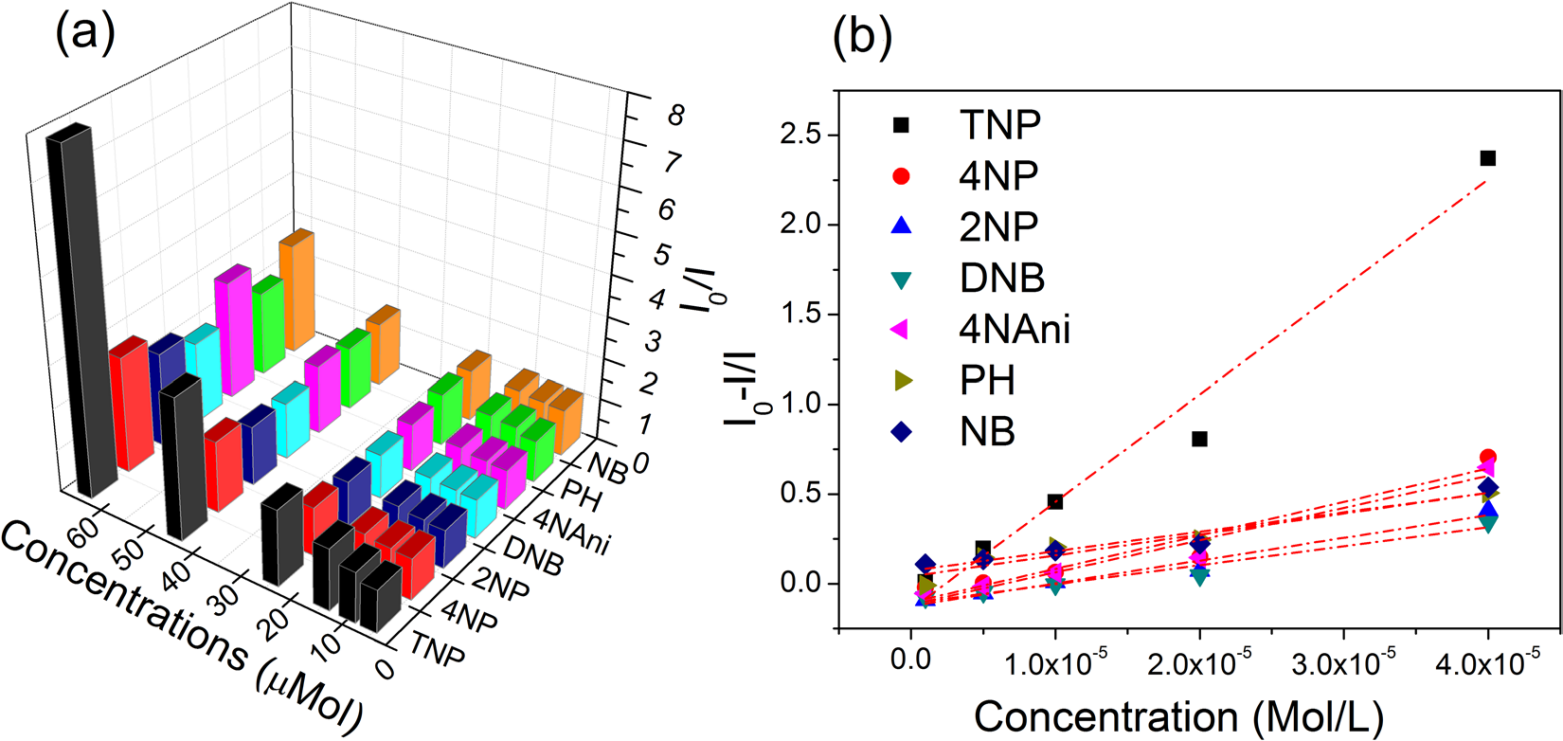
Comparison of quenching efficiency of TNP and other similar nitroaromatics and phenol. (**a**) Rate of quenching of CDs by different analytes at various concentrations; and (**b**) Stern-Volmer plots for different analytes in the concentration range of 0–40 µM. The dotted lines are the linear fits.

In order to be an effective sensor, in addition to being sensitive, the material must be selective. To assay the selectivity of CDs towards TNP sensing, fluorescence quenching titrations were also performed with other nitro-compounds — 4-nitrophenol (4NP), 2-nitrophenol (2NP), dinitro-benzene (DNB), nitroaniline (NAni), phenol (PH) and nitrobenzene (NB).We can see from Fig. 9a, that all the nitro-compounds affect a PL quenching, but the rate of quenching with TNP is by far the maximum. While, 80% PL quenching is affected at 60 µM of TNP addition, the closest competitors like 4NP, 4NAni and NB can cause ~30% or less quenching for the same concentration. The colloidal CDs therefore, exhibit higher selectivity towards TNP compared to other nitro-compounds investigated here.

Quantitative assessment of the quenching efficiencies of different quenchers was performed by standard Stern–Volmer (S-V) method (Supporting Information). The S-V quenching coefficient (*K*_*SV*_), which is a measure of sensitivity towards an analyte, is found to be significantly larger for TNP suggesting dominant selectivity of TNP over others as shown in Fig. 9b. The *K*_*SV*_ values of different analytes provided in Table S2 (Supplementary Information) demonstrate that *K*_*SV*_ of TNP is at least three times or more compared to the other nitro-aromatics.

In recent times, different materials, including many carbon based nano-systems have been investigated for detection of TNP by PL quenching^70–72^. Interestingly, in almost all cases, it has been reported that TNP affects a preferential quenching of luminescence. Mechanisms such as Fröster (fluorescence) resonance energy transfer (FRET), charge transfer, inner filter effect, etc. have been suggested to be responsible for the observed selectivity^73^. In this communication we will refrain from dealing with the mechanism of selective detection. We will simply note that at lower concentrations, the S-V plots of TNP are close to linear (Figure S3a–c) but for higher concentrations, it deviates from linearity and increases almost exponentially (Figure S3d). The linear S-V plots can be attributed to the ground state interactions between the TNP and the CDs, whereas the non-linear S-V plots of TNP suggest that quenching is mainly due to energy and/or electron transfer processes between the analyte and the CDs^74^. It is also observed (Figure S4) that the emission band of the CDs overlaps with the absorption band of the TNP, although the overlap integral is not sufficiently large to unambiguously confirm FRET^75^. At this stage we believe, multiple mechanisms involving the surface groups are involved in selective detection of TNP by the amorphous CDs. A detailed analysis of this phenomenon warrants a separate investigation.

In summary, a simple and inexpensive method to prepare scalable quantities of amorphous CDs and their application in sensing TNP are demonstrated. Carbonization of glucose by ultrasonic waves leads to the formation self-passivated clusters of carbon atoms — the amorphous CDs. The CDs exhibit intense room temperature PL, which closely resemble the luminescent character of their crystalline counterparts. Abundant surface traps and dominant π-π* and n-π* transitions are responsible for excitation dependent PL from the CD colloids. These CDs are found to be excellent candidates for sensitive and selective detection of 2,4,6-TNP. Rapid and intense quenching of PL intensity of the CD colloid was observed due to the addition of TNP, which allowed detection of TNP simply by visual inspection, up to a concentration of 0.5 µM. In general, the CDs can selectively detect TNP over a wide range of concentrations (up to 200 µM) with a detection limit of 0.2 µM, which is one of the best performances reported so far.

## Methods

### Materials

Dextrose (Merck, India), hydrochloric acid (Fisher Scientific), 2,4,6-TNP, 4NP, 2NP, Nitrobenzene, Dinitrobenze, Nitro aniline and Phenol (Loba Chemicals, India). All chemicals were of synthesis grade and used as received without further purification. 18.2 MΩ.cm, Millipore deionised water was used for all synthesis and characterization.

### Preparation of carbon dots

The preparation of amorphous CDs involved simple ultrasonication (Piezo U-Sonic) of a 1:1 (volume ratio) mixture of 1 M dextrose solution and HCl. With increasing time of ultrasonication the solution changed from colorless to brown. After 12 h of sonication the brown colored solution was oven-dried at 80 °C under ambient pressure for 24 hours to obtain a dark brown colored dry powder. This powder was dissolved in DI water and centrifuged at 12000 rpm for 15 min. The light yellowish supernatant was separated and found to contain fine dispersion of water-soluble, amorphous CDs. It was found that on an average 9 gms of dextrose produces 6.5 gms of dry CD powder implying a production yield of 72.2% (Supplementary Information).

### Characterization

Investigation of crystalline property of the synthesized CDs was carried out by XRD using a Brucker D8 advanced diffractometer operating at 40 kV, using Cu *Kα*_1_ (λ = 1.54056 Å) radiation. Structure of the samples was investigated by HR-TEM using a JEOL JEM-2200FS electron microscope equipped with a 200 kV field emission gun and in-column energy filter (Omega) along with Gatan software. The samples were five times diluted prior to deposition and 2 μl of samples were deposited on carbon coated cupper grids for HR-TEM measurements. EELS spectra were collected around the carbon K-edge to understand the chemical bonding and low-energy-loss EELS was performed to extract additional information including mass density. FTIR spectra were recorded using a Bruker, Tensor 27 FTIR spectrometer. XPS measurements were conducted on an Omicron Multiprobe (Omicron NanoTechnology Gmbh., UK) spectrometer fitted with an EA125 (Omicron) hemispherical analyzer. Monochromatic Al-K_*α*_ source operated at 150 W was used and the pass energy of the analyzer was kept at 40 eV. A low-energy electron gun (SL1000, Omicron) with a large spot size was used for sample neutralization. The voltage of the electron gun was fixed at −3 V. UV-visible spectroscopy was performed using a JASCO V-750, UV-VIS spectrophotometer. Steady state PL property of different samples was investigated using a Horiba JobinYvon, Fluorolog-3 (Nanolog) spectrofluorometer (model FL3–11) fitted with a 450 W xenon lamp source, photomultiplier tube detector and single grating monochromator. During observation, entry and exit slit widths were kept at 1 nm and the integrating time was 0.5 s. PL yields of the samples were estimated by recording the PL spectra using an integrating sphere following the method proposed by Pålsson and Monkman^33^, and de Mello *et al*.^34^. The integrating sphere was mounted inside the Fluorolog-3 spectrofluorometer and the samples were mounted into the holder inside the sphere. The measured spectra were background corrected by recording a blank data.

## Electronic supplementary material

Supplementary Information
