# Supplementary material for: Amorphous Carbon Dots and their Remarkable Ability to Detect 2,4,6-Trinitrophenol
Source: Sci Rep. 2018 Jun 27;8:9770. doi: 10.1038/s41598-018-28021-9 (PMC6021439; doi:10.1038/s41598-018-28021-9)
Supplement: Supplementary file 1 — Supplementary Information [file 41598_2018_28021_MOESM1_ESM.docx]

**Supplementary Information**

**Amorphous Carbon Dots and their Remarkable Ability to Detect 2, 4, 6-Trinitrophenol**

**Abu Bakar Siddique**^1,^***, Ashit Kumar Pramanick**^2^**, Subrata Chatterjee**^1^**, and**

**Mallar Ray**^1,^*

^1^Dr. M. N. Dastur School of Materials Science and Engineering, Indian Institute of Engineering Science and Technology, Shibpur, PO. Botanic Garden, Howrah, 711103, India

^2^Materials Science Division, National Metallurgical Laboratory, Jamshedpur, 831007, India

*mray@matsc.iiests.ac.in

**1. The performance of the CDs in detecting TNP in comparison to other reported materials**

**Table S1.** Comparative performances of TNP sensor materials:

| **Material/s** | **Method** | **Detection limit** | **Selectivity** | **Year** | **Remarks** | **Reference** |
| --- | --- | --- | --- | --- | --- | --- |
| **Amorphous**  **Carbon**  **Dots** | PL quenching | 0.2 μM | Yes | 2017 | Non-toxic, easy bulk preparation at low cost | Present work |
| Metal−  Organic Framework | PL quenching | 5 μM | Yes | 2017 | Toxic (Cd), expensive, sensitivity lesser than present work | Dong *et. al.* *Cryst. Growth Des.* **2017** (Nov 27; web)  **DOI:**10.1021/acs.cgd.7b01430 |
| N-doped graphene quantum dots | PL quenching | 0.92 μM | Yes | 2017 | Relatively complex preparation, sensitivity lesser than present work | Kaur *et. al*. *Spectrochim. Acta* **2017,** *180* 37–43 |
| Napthalene based complex prepared from 3,4-Diaminofurazan and  2-hydroxy-1-naphthaldehyde | PL quenching | 0.005 μM using nonlinear titration relation | Dual sensing of TNP and TNT | 2017 | Using non-linear titration relation to find detection limit would drastically lower the sensitivity of the present as well as other reported materials | Zang *et al*. Talanta  2017, *166*, 228-233 |
| Crystalline carbon dots and polypyrrole | Electrical detection | 0.14 μM | Yes | 2016 | Complex and expensive preparation and set up, sensitivity marginally better | Pal *et. al.* *ACS Appl. Mater. Interfaces* **2016**, *8*, 5758–5762 |
| Carbon quantum dots functionalized with amines | PL quenching | 0.2 μM | Yes | 2016 | Similar performance but functionalized crystalline carbon dots | Campos *et al.* Carbon. **2016,** *106,* 171-178 |
| 2D metal-organic frameworks | PL quenching | 0.27 μM | Yes | 2016 | Complex preparation, expensive, sensitivity marginally less than present work | Rachuri *et al*. *Dalton Trans.* **2016**, *45*, 7881–7892 |
| Cd based metal-organic framework | PL quenching | 2 µM | Yes | 2016 | Toxic (Cd), expensive, sensitivity much less than present work | Wang *et al*. *Inorg. Chem. Commun*. **2016**, *68*, 45–49. |
| Lysozyme-capped CdS quantum dots | PL quenching | 0.1 μM | Yes | 2015 | Toxic (Cd), relatively more expensive, sensitivity better | Na *et al*.  *RSC Adv*. **2015**, *5*, 51428–51434 |
| Glucopyranosyl-1,4-dihydropyridine | PL quenching | 0.94 μM | Yes | 2015 | Expensive chemicals, sensitivity lesser than present work | Pinrat *et. al.* *Analyst***. 2015**, *140*, 1886 -1893 |
| Donor–acceptor fluorescent molecule composed of anthracene and 1,8-Naphthalimide units | PL quenching | 2.59 μM | Yes | 2015 | More expensive and complex preparation; sensitivity lesser than present work | Ye *et. al.* *Sens. Actuators B* **2015,** *210*, 566–573 |
| Europium (III) Metal–Organic Framework | PL quenching | 4.98 μM | Yes | 2014 | Complex and expensive preparation, sensitivity lesser than present work | Song et al. *Adv. Funct. Mater.* **2014**, *24*, 4034-4041 |

**2. Estimation of the density of the CDs from the plasmon energy peak in EELS spectrum:**

The plasmon peak position (*E_p_*) is related to the density of valence electrons (*n_e_*)^1^ by:

 (S1)

where, *ε*_0_ is the vacuum dielectric constant and *m** is the electron effective mass, assumed to be:

*m*^*^=0.87*m* (S2)

*m* being the free electron mass.

The mass density is derived from the valence electron density, *n_e_*, by assuming that carbon contributes four valence electrons. Therefore, the mass density, *ρ*, turns out to be:

 (S3)

where, *M_C_* is the molar mass of carbon (12 g/Mol) and *N_A_* is the Avogadro number.

**3. Calculation of PL Quantum yield:**

The PL QY was subsequently determined according to the method outlined by de Mello.^2^ In this approach PL QY is given by:

Photons absorbed = *∫I(λ)dλ* (Blank cuvette in integrating sphere) - *∫I(λ)dλ* (Sample in integrating sphere); at the excitation of 330 for small window of emission spectrum (325 – 335 nm)

Photons emitted = *∫I(λ) dλ*(Sample) - *∫I(λ) dλ*(Blank) ; at the excitation of 330 for full emission spectrum range (350 – 600nm)

$$PLQY= \frac{Photons Emitted}{Photons Absorbed}$$

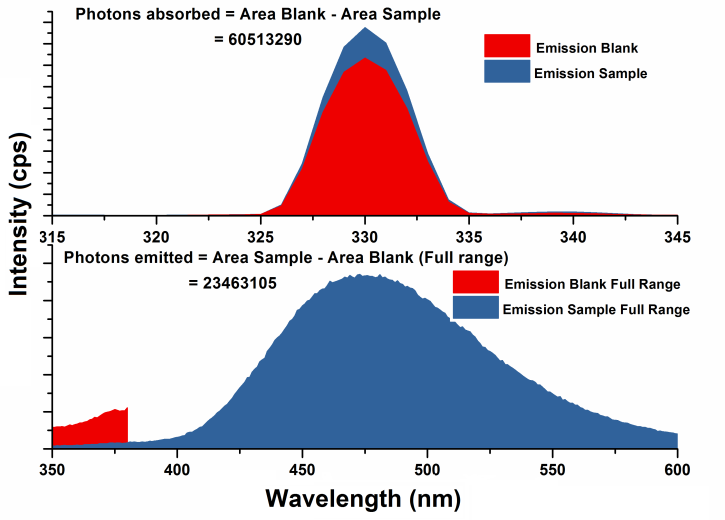


**Figure S1.** Total emission and absorption of samples for determination of PL quantum yield using an integrating sphere

**4. Variation of PL intensity with TNP additions in CDs:**


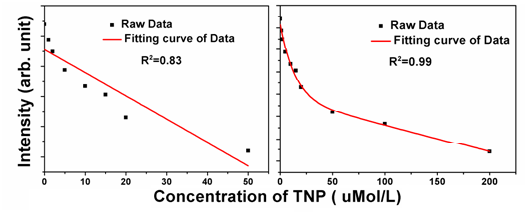


**Figure S2.** Variation of maximum PL intensity with addition of different concentrations of TNP to the colloidal CDs – (a) the linear variation up to 50 µM addition, and (b) the non-linear quenching beyond 50 µM and up to 200 µM

**5. Stern–Volmer Plots and Quenching Efficiencies:**

The standard Stern–Volmer (S-V) equation was used:

$\frac{I_{0}(\lambda)}{I(\lambda)}=K_{SV}\left(\lambda\right)\left[Q\right]+1$ (S4)


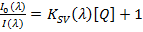


Where, *I_0_*(*λ*) and *I*(*λ*) are respectively the PL intensities at a specified emission wavelength *λ* in the absence and presence of quencher, [*Q*] is the quencher concentration and *K_SV_*(*λ*) is the Stern−Volmer quenching constant at emission wavelength *λ*.

The S-V plots for the TNP in various concentration ranges are shown in Figure S3 (a)-(d). Quantitatively, of course, the fluorescence quenching by TNP is distinct up to much lower concentration (0.2 µM) as evidenced from the S-V plot in the calibrated curve of linear concentration range of 0 to 20 µM, shown in the Figure S3a. The slope of the curve gives the *K_SV_*(*λ*) value. Higher the value of *K_SV_*(*λ*), more is the sensitivity towards that analyte. We see that *K_SV_* values for TNP are significantly larger than those of the other nitroaromatic compounds and phenol, suggesting dominant selectivity of TNP. The *K_SV_*(*λ*) values of different analytes are tabulated in Table S1.

**Table S2:** Stern- Volmer constant, *K_SV_*(*λ*) values of different analytes:

| **Analytes** | **TNP** | **4NP** | **2NP** | **DNB** | **4NAni** | **PH** | **NB** |
| --- | --- | --- | --- | --- | --- | --- | --- |
| **K_sv_ (M^-1^ )** | 59921 | 18678 | 12671 | 10605 | 17974 | 11663 | 10851 |

The fluorescence quenching type could be analyzed by Stern–Volmer equation, the curve would fitted to be linear if the quenching type is single (static) or fitted to an upward curvature if it is combined (dynamic).^3, 4^


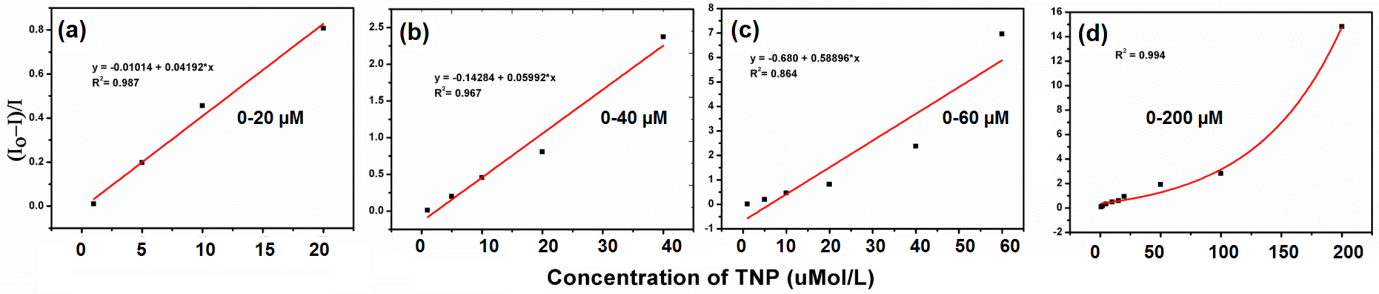


**Figure S3**. Stern-Volmer plots of PL quenching at different concentrationranges of TNP addition


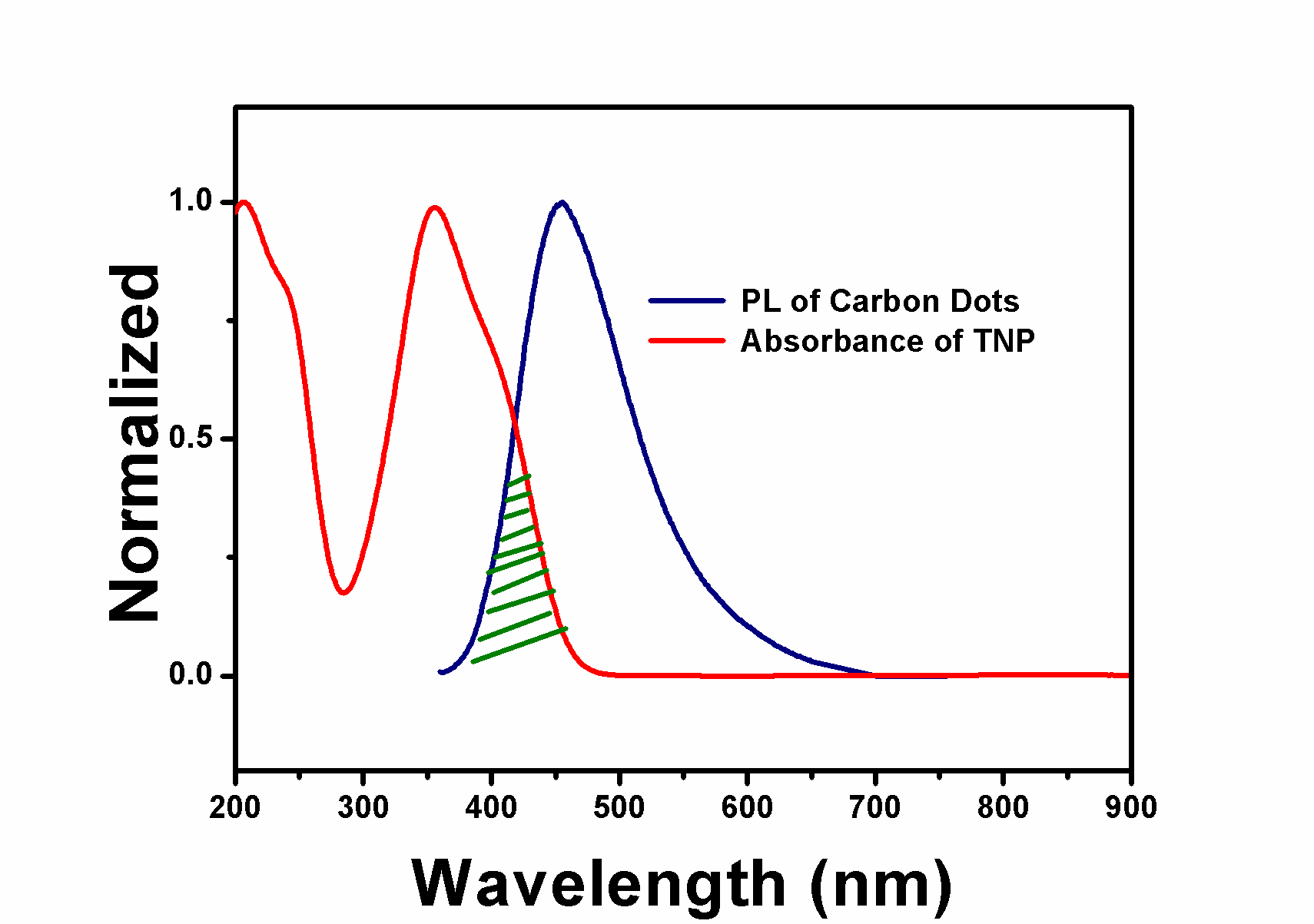


**Figure S4.** Overlap of the fluorophore (CDs) emission band and quencher (TNP) absorption spectrum

The standard method to conclude if FRET is the dominant mechanism responsible for quenching is to find out the extent of overlap of the fluorophore emission and quencher absorption spectrum.^5^ On the other hand, the relative position of LUMO energy of an analyte with respect to the LUMO (or conduction band edge) of a sensor material is considered to determine the direction of electron transfer upon photoexcitation. The decreasing LUMO energy levels of nitro aromatics represent how easily excited electrons are transferred from the higher energy LUMO of the CDs to the lower energy LUMO of electron-deficient nitro compound. Although, TNP has the lowest LUMO energy level, the quenching efficiency of all nitro-compounds is not well in agreement with this band energy difference. This suggests that electron transfer is unlikely to be the dominant mechanism. We also see from Figure S4 that the overlap of the CD emission and TNP absorption is not very large. Hence, it is difficult to believe that FRET is primarily responsible. From the current results it is difficult to assert on any particular mechanism. We have taken up detailed investigation to resolve the issue of the mechanism of selective detection.

**6. Production yield of the CDs:**

The method described in this work produces CDs in scalable quantities. The production yield determined simply using — [Oven dried solid CDs/Total precursor material, i.e. Dextrose] x 100 is found to be 72.2% (average value).

**Table S3. Comparison of production yield of CDs with other reported works**

| Sl No. | References | Yield |
| --- | --- | --- |
| 1. | Yang et al. *J. Colloid and Interf. Sci.* **492**, 1-7 (2017) | 41.2% |
| 2. | Li et al. *J. Mater. Chem. B* **5**, 1935—1942 (2017) | 25.8 – 66.7% |
| 3. | Sun et al. *Phys. Chem. Chem. Phys.* **15**, 9907—9913 (2013) | 63±7% |
| **4.** | **Present work** | **72.2%** |

1. Ferrari, A. C.; Libassi, A.; Tanner, B. K.; Stolojan, V.; Yuan, J.; Yuan, J.; Brown, L. M.; Rodil, S. E.; Kleinsorge, B.; Robertson, J. Density, sp^3^ Fraction, and Cross-Sectional Structure of Amorphous Carbon Films Determined by X-Ray Reflectivity and Electron Energy-Loss Spectroscopy. *Phys. Rev. B* **2000**, *62*, 11089–11103.
2. Mello, J. C. de; Wittmann, H. F.; Friend, R. H. An Improved Experimental Determination of External Photoluminescence Quantum Efficiency. *Adv. Mater*. **1997**, *9*, 230–232
3. Zhu, X.; Gong, A.; Wang, B.; Yu, S. Study on the Interaction of Tropisetron Hydrochloride and L-Tryptophan by Spectrofluorimetry and Its Analytical Application. *J. Lumin.* **2008**, *128*, 1815–1818.
4. Kandagal, P. B.; Ashoka, S.; Seetharamappa, J.; Shaikh, S. M. T.; Jadegoud, Y.; Ijare, O. B. Study of the Interaction of an Anticancer Drug with Human and Bovine Serum Albumin: Spectroscopic Approach. *J. Pharm. Biomed. Anal .***2006**, *41*, 393–399.
5. Rong, M.; Lin, L.; Song, X.; Zhao, T.; Zhong, Y.; Yan, J.; Wang, Y.; Chen, X. A Label-Free Fluorescence Sensing Approach for Selective and Sensitive Detection of 2,4,6-Trinitrophenol (TNP) in Aqueous Solution Using Graphitic Carbon Nitride Nanosheets. *Anal. Chem.* **2015**, *87*, 1288–1296.
